# Understanding the links between hearing impairment and dementia: development and validation of the Social and Emotional Impact of Hearing Impairment (SEI-HI) questionnaire

**DOI:** 10.1007/s10072-020-04492-5

**Published:** 2020-06-10

**Authors:** Jenna Littlejohn, Daniel Blackburn, Annalena Venneri

**Affiliations:** 1 Department of Neuroscience, University of Sheffield, Sheffield, UK; 2 Manchester Centre for Audiology and Deafness, University of Manchester, Manchester, UK

**Keywords:** Hearing impairment, Psychosocial, Validation, Questionnaire

## Abstract

**Background:**

The links between hearing impairment (HI) and dementia have been well documented, but factors mediating this relationship remain unknown. Major consequences of HI are social and emotional dysfunction, and as the risk of dementia increases linearly with the severity of HI, it is plausible that socio-emotional difficulties may play a role in this association.

**Objective:**

The aim of this study was to develop and validate a tool to analyse levels of hearing-related disability, to investigate ultimately whether subjective disability contributes to risk of cognitive impairment compared with hearing thresholds alone.

**Methods:**

Development and validation of the questionnaire, the Social and Emotional Impact of Hearing Impairment (SEI-HI), was conducted in four phases: (1) content; (2) scoring and outcomes; (3) validation; (4) feasibility in a sample of people with cognitive impairment.

**Results:**

Considerable evidence was found for the internal and external reliability of the tool with high construct validity, concurrent validity and test-retest values of the SEI-HI questionnaire. A feasibility check on 31 patients with mild cognitive impairment or dementia showed the SEI-HI questionnaire was easy to administer and well-received.

**Conclusion:**

The SEI-HI questionnaire is a relevant instrument to assess hearing-related disability which can be used in people with cognitive decline to assess further impact on risk of developing dementia.

**Electronic supplementary material:**

The online version of this article (10.1007/s10072-020-04492-5) contains supplementary material, which is available to authorized users.

## Introduction

Hearing impairment (HI) is one of the most common disabilities of the ageing population affecting over 466 million people worldwide [1]. One of the main debilitating features of HI is communication difficulties which affect personal relationships and leads to withdrawal from social situations [2]. HI may be particularly disadvantageous for older adults who have not developed skills to cope with communication difficulties [3], and as a consequence, HI in the elderly is associated with reduced quality of life, depression, functional decline, lowered self-esteem and social isolation [4–9].

HI is common in people living with dementia [10], and is one of nine potentially modifiable risk factors [11]. However, the *mechanism* linking HI to dementia remains to be elucidated. One theory is HI may indirectly increase the risk via psychosocial pathways [12]; people with HI are more likely to feel lonely and socially isolated [2], and social and emotional dysfunction are independently associated with the risk of dementia. Loneliness has shown to accelerate levels of cognitive impairment [13] and risk of developing Alzheimer’s disease dementia [14]. People with poor social networks are 60% more likely to develop dementia compared with those with good social networks [15].

The International Classification of Functioning, Disability and Health, an international standard set by the World Health Organisation [16], recognises that many factors are responsible for self-perception of disability. In the case of HI, for any given auditory threshold, there will be a large variability in the level of associated disability [17]. Many factors must be considered, such as whether the individual is socially active and whether they use hearing aids and additional family support, as well as personal factors including age, background, and lived experiences. For this reason, it should not be assumed that a person with a mild HI would have a milder disability compared with someone with a more severe HI.

Evidence to support this theory comes from hearing aid studies. Management of HI by hearing aids improves quality of life [18–20]. Also, albeit inconsistently, the use of hearing aids has been shown to improve short-term cognitive performance [21], and attenuate the increased risk of cognitive decline associated with HI [22].

Tools have been designed to measure hearing-related disability, but are not appropriate for this use for many reasons; they are either now outdated, no longer culturally or technologically relevant, have not been validated on people living with cognitive impairment, or were designed for a different purpose (i.e. as a measure of before and after for hearing aid rehabilitation). The aim of this study was to design and validate a short, easy to administer, culturally relevant questionnaire to measure the social and emotional impact of HI. This tool could then be used to investigate the indirect psychosocial pathway hypothesis linking HI to increased rates of cognitive decline and dementia. This may have a major impact on future public health as a case for more aggressive treatment of HI and social isolation, to reduce the burden and onset of cognitive impairment.

## Methods

Development and validation of the Social and Emotional Impact of Hearing Impairment (SEI-HI) questionnaire will be described in four phases. For ease of understanding, demographic characteristics of participants involved in each phase are described in Table 1. Ethical approval was obtained from NRES Committee North East–Newcastle and North Tyneside (ref 170445, 15/NE/0152). All participants gave their informed written consent.

**Table 1:** Participants characteristics involved in the four phases

	(1) Content	(2) Scoring	(3) Validation	(4) Feasibility
Number	80	120	95	31
Age (SD)	63.55 (11.85)	57.82 (12.19)	59.13 (12.67)	67.94 (9.67)
Male/female	28/52	36/84	36/59	20/11
YOE (SD)	14.38 (3.52)	15.63 (3.04)	15.47 (3.28)	12.00 (2.35)
NH/HI	45/35	120/0	57/38	12/19

Age and YOE are reported as mean number of years, with standard deviation (SD) in brackets

*YOE* years of education, *NH* normal hearing, *HI* hearing impairment

### Phase 1: Content

Pure tone average (PTAv) thresholds in the better hearing ear were recorded for frequencies at 500, 1000, 2000 and 4000 Hz for each participant. They were then asked to complete the 25-item hearing handicap inventory for the elderly (HHIE) [23], responding with ‘yes’, ‘sometimes’ or ‘no’ to each question and to expand on their responses verbally, which were noted as ‘free feedback’.

To establish the criteria that were the most commonly reported to be problematic, quantitative responses to each item on the HHIE were analysed and each item was given a rank (from 1 to 24) depending on scoring frequency. For each participant, items with positive responses, (i.e. a response of ‘yes’ or ‘sometimes’) were given a score of 1, which was tallied and the 10 highest scoring items were identified as the most common and pertinent scenarios. The consultation allowed further adaptations of the scenarios to suit the influence of HI on our sample and more generally to devise an assessment procedure suitable for a variety of individuals, with clinical and non-clinical samples. Together, this evidence informed the creation of the 14-item SEI-HI questionnaire (Online resource 1).

### Phase 2: Scoring and outcomes

Free feedback from Phase 1 helped to develop the appropriate scoring rating for the questionnaire. Respondents from Phase 1 revealed the need for an ‘in between’ measure as in certain situations, responses were not as clear cut as ‘yes’ or ‘sometimes’. Participants stated: “It is not never, but not as much as sometimes” or “It’s more than sometimes but I wouldn’t say that it was ‘yes’ a definite issue for me.” For this reason, the 14 items were formatted using a five-point Likert scale. According to Millers law, the limit on the amount of information that can be held in our working memory at any one time is 7 items, plus or minus 2 [24]; therefore, the five-point scale was selected as it would offer enough choice and still be manageable for the participants with varying levels of cognition.

One of five responses can be given for each question: 1 = never, 2 = occasionally, 3 = half the time, 4 = frequently, 5 = always. Responses from the 14 questions are summed to develop a raw overall score between 14 and 70, which is then converted into an overall percentage disability by simple calculation:

$$ SEI- HI\  Disability\ \left(\%\right)=\left(\left(\frac{score}{14}\right)-1\right)\times 25 $$

The higher the percentage, the more restricted a person feels. One hundred and twenty participants with normal hearing thresholds (Table 1) were asked to complete the SEI-HI questionnaire according to written instructions, following hearing screening to ensure normal hearing levels (classified as PTAv < 25 dB.)

### Phase 3: Psychometric validation

To verify psychometric validity and reliability of the SEI-HI questionnaire, face validity, internal consistency, concurrent validity, reliability and the role of experimenter bias of the SEI-HI questionnaire were explored in 95 participants with mixed hearing thresholds (Table 1).

#### Face validity

Face validity was assessed by a subsample of 10 participants, chosen as they all had experience of working with or supporting people with HI. Five participants had HI ranging from mild to severe, and five had normal hearing. Participants were asked (1) whether they felt the instructions on the questionnaire were clear and easy to understand; (2) if all of the questions were clear and easy to understand; and (3) whether the questions were relevant to the HI population.

#### Internal consistency

Internal consistency and reliability of the scale was measured using Cronbach’s alpha coefficient. A coefficient of .7 or .8 is generally regarded as having high internal consistency.

#### Concurrent validity

Concurrent validity was examined using Spearman’s Rho correlation coefficient to observe similarities between outcomes on the SEI-HI questionnaire and two other questionnaires, the HHIE and Self-Assessment of Communication (SAC) [25].

#### Test-retest reliability and experimenter bias

All participants were asked whether they would be available for repeat testing, and a convenience subsample comprising of the first 35 was selected for retesting over a 4–8-week period. In this subsample, there were 15 males and 20 females with mean age of 57.06 (SD = 13.01).

An intra-class correlation coefficient was used to examine the degree of correlation and agreement between the scores at the different time points (T1 and T2). Intra-class correlation coefficient estimates and their 95% confidence intervals were calculated based on a single-rating, absolute-agreement, 2-way mixed-effects model. Subsequently, a related-samples Wilcoxon Signed-Rank Test was undertaken to compare differences between the two time points. Fifteen (43%) of the retest participants were followed up by a different examiner to control for experimenter bias and 95% confidence intervals were inspected.

### Phase 4: Feasibility

To ensure validity in a sample of people with cognitive impairment, participants with varying levels of cognitive impairment were asked to complete the SEI-HI questionnaire, with support from the experimenter. There were 18 patients with mild cognitive impairment and 13 with dementia (AD (*n* = 7); dementia with Lewy bodies (*n* = 2); frontotemporal dementia (*n* = 3); and corticobasal degeneration (*n* = 1)).

## Results

### Phase 1: Content

Scoring frequency for each item of the HHIE is recorded in Table 2. Free feedback allowed discussion around wording of the questions, and modification of the top 10 ranking situations, described in Table 2, for inclusion into the SEI-HI questionnaire.

**Table 2:** Items on the HHIE ranked according to positive response

Question on HHIE	Y	S	Total	Rank
[S15] Does a hearing problem cause you difficulty when listening to the TV or radio?	26	15	41	1
[S8] Do you have difficulty hearing when someone speaks in a whisper?	26	13	39	2
[S6] Does a hearing problem cause you difficulty when attending a party?	27	10	37	3
[S21] Does a hearing problem cause you difficulty when in a restaurant with relatives or friends?	21	10	31	4
[E25] Does a hearing problem cause you to feel left out when you are in a group of people?	20	10	30	5
[E20] Do you feel that any difficulty with your hearing limits or hampers your personal or social life?	19	6	25	6
[E5] Does a hearing problem cause you to feel frustrated when talking to members of your family?	14	10	24	7
[E9] Do you feel handicapped by a hearing problem?	17	4	21	8
[E7] Does a hearing problem cause you to feel stupid or dumb?	10	11	21	9
[E4] Does a hearing problem make your irritable?	11	9	20	10
[S1] Does a hearing problem cause you to use the phone less often than you would like?	18	1	19	11
[S3] Does a hearing problem cause you to avoid groups of people?	14	4	18	12
[E2] Does a hearing problem cause you to feel embarrassed when meeting new people?	10	8	18	13
[E17] Does any problem or difficulty with your hearing upset you at all?	9	9	18	14
[S10] Does a hearing problem cause you difficulty when visiting friends, relatives, or neighbours?	10	7	17	15
[S23] Does a hearing problem cause you to listen to the TV or radio less often than you would like?	14	1	15	16
[E18] Does a hearing problem cause you to want to be by yourself?	7	7	14	17
[E14] Does a hearing problem cause you to have arguments with family members?	6	8	14	18
[E24] Does a hearing problem cause you to feel uncomfortable when talking to friends?	7	6	13	19
[E12] Does a hearing problem cause you to be nervous?	7	4	11	20
[S19] Does a hearing problem cause you to talk to family members less often than you would like?	5	3	8	21
[E22] Does a hearing problem cause you to feel depressed?	3	4	7	22
[S13] Does a hearing problem cause you to visit friends, relatives, or neighbours less often than you would like?	4	0	4	23
[S16] Does a hearing problem cause you to go shopping less often than you would like?	2	2	4	24
[S11] Does a hearing problem cause you to attend religious services less often than you would like?	2	1	3	25

*Y* yes, *S* sometimes

#### Hearing aids

The use of hearing aids was a common theme that came up during free feedback. The HHIE instructs respondents to answer how they would feel if they were not wearing their hearing aids. Participants who wore hearing aids felt this did not make the questions relevant to their current situation. For instance, S10 (*Does a hearing problem cause you difficulty when visiting friends*, *relatives or neighbours*?). The comments were ‘well it would if I weren’t wearing my hearing aids, but I always wear them and don’t have any difficulty.’ For this reason, it was imperative to ensure that the instructions on the SEI-HI questionnaire were clear for participants to answer how they are *currently* feeling regarding their hearing situation. This also prompted inclusion of a question regarding hearing aids; participants are asked if they wear hearing aids, and if so, (a) on an average day, how long do they wear the hearing aids for and (b) what is their overall satisfaction with their hearing aids (on a scale of 1 to 5). As many intrinsic and extrinsic factors may affect satisfaction, a single question was chosen to encompass overall satisfaction, aiming to identify an evaluation of hearing aids against their personal expectations.

#### Phrasing of questions

Another common theme to emerge was that participants stated hearing problems do cause difficulties in the scenario, but only under certain circumstances. For example, S21 (*Does a hearing problem cause you difficulty when in a restaurant with relatives or friends*?), one respondent said ‘It can do, but it depends on the time of day, where I am or who I am with’. For this reason, questions on the SEI-HI questionnaire were phrased to take this into account and ask ‘How often...’ rather than ‘Do you...’.

#### Modifications to themes

Free feedback allowed for adaptation of the most commonly reported difficulties to the HHIE, to make situations more relevant. For example, many participants with HI stated sometimes having difficulty listening to the TV or radio, but this difficulty is dependent on the channel, programme or external features (e.g. background noise). These difficulties do not stop them from watching it or cause watching it less often, as with the use of subtitles, hearing aids or assistive listening devices, they can continue to enjoy programmes. So, as they have adapted new habits, they do not necessarily feel disadvantaged or restricted by this. Another example was from E2 (‘*Does a hearing problem cause you to feel embarrassed when meeting new people*?’) When answering this question, it was commonly reported that participants did not feel embarrassed when *meeting new people*, but embarrassment when losing track of conversations or not being able to answer questions in a social or work scenario.

#### Inclusion of new topics

Free feedback associated with S6 (‘*Does a hearing problem cause you difficulty when attending a party*’). Participants commonly stated that it is not the fact that it is a party; it is any social situation in which there are groups of people or excessive noise. Participants reported a big distinction between difficulty hearing during a one to one conversation compared with being in a group of people, which was notably harder, and even more difficult in a noisy environment. Few participants noted that as their HI progressed, even conversing with one person was becoming more difficult and led to the complete avoidance of party situations where there would be both groups and noise. As a consequence, the SEI-HI questionnaire includes specific questions around difficulty with one other person, small groups of people and noisy situations, as well as avoidance at parties.

### Phase 2: Scoring and outcomes

**Fig. 1:**
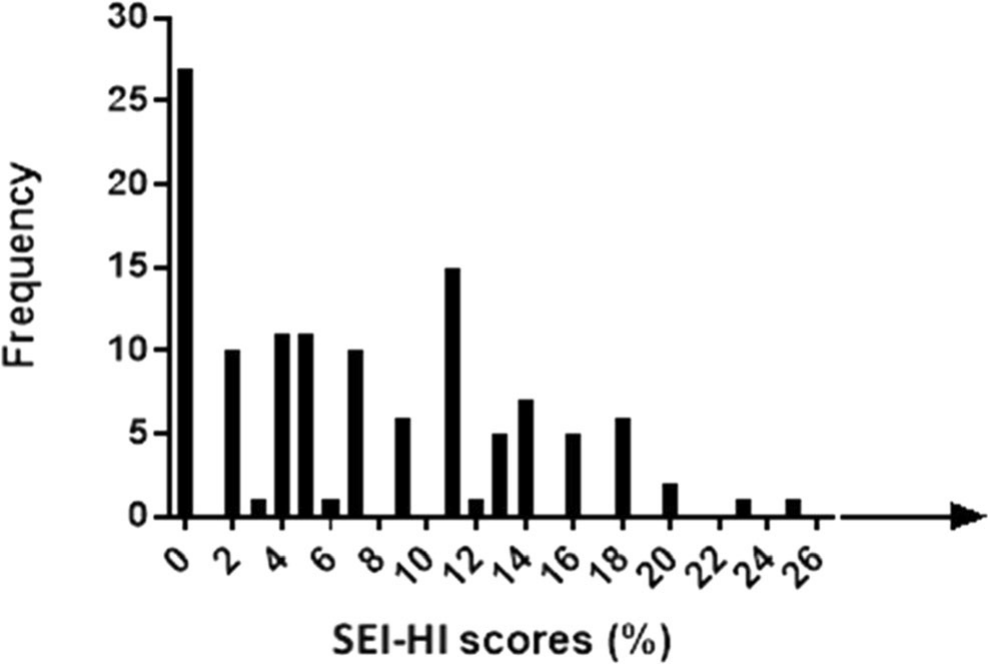
Distribution of SEI-HI questionnaire scores for participants with normal hearing thresholds

As PTAv confirmed normal hearing thresholds (of < 25 dB) for all participants, they were included in the analysis. As expected in a test assessing hearing functioning in people with normal hearing, the distribution of scores showed a positive skew towards the negative end (Fig. 1). The modal overall score was 0; the median score was 5.5, IQR = 9. The highest score from the participants was taken as the clinical cut-off point to ensure minimisation of false positive and false negative scores. According to this, a score < 25% portrays no dysfunction, with > 25% classified as self-perceived social and emotional hearing disability. The larger the percentage, the higher along the functioning-disability continuum the individual feels.

### Phase 3: Psychometric validation

#### Face validity

All 10 participants were in agreement that the instructions, and each question was clear, easy to understand and relevant.

#### Internal consistency

There is a high degree of internal consistency of the scale as illustrated by an overall *α* = .957. Individual items positively correlated with each other, ranging from *r* = 0.64 to 0.87. The reliability of the scale would not be improved by removing any of the items in the questionnaire.

#### Concurrent validity

Scores on the SEI-HI questionnaire were significantly correlated with scores on the HHIE and SAC, representative of high concurrent validity, as shown in Table 3.

**Table 3:** Concurrent validity of SEI-HI questionnaire

	SAC	HHIE
SEI-HI	0.900* [0.790, 0.971]	0.910* [0.862, 0.943]

*p < 0.01. [95% CIs reported in brackets]

*HHIE* Hearing Handicap Inventory for the Elderly, *SAC* Self-Assessment of Communication, *SEI-HI* Social and Emotional Impact of Hearing Impairment

#### Test-retest reliability and experimenter bias

There was a strong positive correlation between participants scores on the SEI-HI questionnaire at time 1 and time 2 (4 to 8 weeks later), ICC = .905, *p* < .001, 95% CI [.812, .952] indicating a good test-retest reliability. There was not a statistically significant change in SEI-HI questionnaire scores between time 1 and time 2 (*Z* = − .216, *p* = .829).

The presence of examiner bias was excluded, as the difference between test-retest correlations for Examiner 1, *r*_S_ = .890, *p* < .001, 95% CI [0.677, 0.986], and Examiner 2, *r*_S_ = .737, *p* = .002, 95% CI [0.235, 0.931], was not significantly different.

### Phase 4: Feasibility

All participants with MCI and dementia were able to complete the SEI-HI questionnaire with no difficulty or reported issues, with support of the experimenter. Clinical characteristics, outlined in Table 4, show participants scores on measures of dementia severity. SEI-HI questionnaire scores ranged from 0 to 89, where 14 people reported hearing-related disability (9 MCI and 5 dementia). Table 1 reported 19 participants from Phase 4 to have measured HI, meaning 74% of these reported hearing-related disability. This proportion is similar to that found for participants from Phase 3, where 79% of participants with HI reported hearing-related disability on the SEI-HI questionnaire.

**Table 4:** Phase 4 sample clinical characteristics

	Range	Mean (SD)	Median
MMSE	9–30	24.31 (5.03)	25
CDR	0.5–2	0.72 (0.41)	0.5
ADL	2–6	5.12 (1.02)	6
IADL	1–8	6.59 (2.32)	8

Clinical data shown here were only available for 29 of the 31 participants

*ADL* Activities of Daily Living, *CDR* Clinical Dementia Rating Scale, *IADL* Instrumental Activities of Daily Living, *MMSE* Mini-Mental State Examination

## Discussion

The SEI-HI questionnaire has demonstrated a high level of reliability and validity as a measure of current psychosocial impact of hearing-related disability in older adults.

Internal consistency was demonstrated by strong Cronbach *α* scores. Due to the spectrum of disability associated with HI, despite the high *α*, all items were included to ensure the breadth of relevant questions to maximise clinical potential.

Due to individual differences in many factors including lifestyle, attitudes, comorbid health conditions, and available support networks, it is logical to assent that not any two people with the same levels of HI will be affected in the same way [17, 26]. Because of this, there is no gold standard criterion for objectifying subjective responses to HI, and therefore we were unable to measure criterion validity. However, in cases where this is not suitable, measuring construct validity is adequate [26, 27].

The test-retest reliability of the SEI-HI questionnaire is very satisfactory at ICC = 0.905. As on average a 6-week timescale had passed, it can be assumed with reasonable certainty that participants would not remember their previous scores and thus the coefficient has not been inflated as a result of the retesting procedure. Any minor changes could be reflective of changes in circumstance or attitudes towards the disability or irrelevant temporal factors, such as mood, which may cause a fluctuation in scores over time [28].

Altogether, this lends support for the use of the SEI-HI questionnaire, not only as a cross-sectional instrument to measure current subjective hearing disability, but to be also used for longitudinal purposes. Due to the strong correlation, small standard error and no evidence of experimenter bias, it can be expected that changes over time are as a result of intervention rather than experimental error [29]. Continuing research remains to evaluate the sensitivity to change of the SEI-HI questionnaire, for a more valuable longitudinal measure, which could then monitor the effects of audiological rehabilitation on the impact of HI to dementia.

Although the development of the SEI-HI questionnaire is based upon the scenarios reported in the HHIE, it aims to measure slightly different aspects than the HHIE and SAC, and it is promising to see the significant correlation between scores on the SEI-HI questionnaire and these measures, supporting the specificity of the SEI-HI questionnaire. This high specificity means that it does not allow for cross-condition comparisons. For example, the socio-emotional difficulties measured are due to common problems in relation to HI, and not suitable for measuring quality of life affected by other conditions or disabilities. Similarly, although the SEI-HI questionnaire has shown high validity and reliability in our sample, it may have limited uses in other cultures and thus may not be generalisable to non-English speaking countries. However, using the principles upon which the scale was designed and validated would allow for translation and validation into other languages. Participants recruited for Phase 4, with MCI to mild dementia, were able to complete the SEI-HI questionnaire with support from the experimenter. This has not been designed as an informant questionnaire, due to the sensitive and subjective nature of hearing disability. In cases with a more severe cognitive impairment, it will be left to the clinicians’ or researchers’ judgement to include carer-responses to aid answering these questions.

Given the clinical importance of investigating this association, it is essential to have a specific, valid and reliable questionnaire to compute current levels of hearing functioning. To the best of our knowledge, the SEI-HI questionnaire is the first validated instrument to measure current levels of subjective hearing disability in recent years. To conclude, this study has shown that the SEI-HI questionnaire is a favourable and relevant instrument to assess current levels of subjective hearing disability regardless of hearing threshold. It can be used with confidence to control for subjective levels of disability in people with varying levels of HI, to assess further the risk of cognitive decline. The use of the SEI-HI questionnaire would help to determine whether the social and emotional impacts of HI have more of an influence on the risk for dementia, in addition to hearing thresholds alone.

## Electronic supplementary material

ESM 1 (DOCX 23 kb)

## References

[1] WHO (2019) Deafness and hearing loss. [cited 2020 30.01.20]; Available from: https://www.who.int/news-room/fact-sheets/detail/deafness-and-hearing-loss

[2] Strawbridge WJ, Wallhagen MI, Shema SJ, Kaplan GA (2000). Negative consequences of hearing impairment in old age: a longitudinal analysis. Gerontologist.

[3] Livneh H, Wilson LM (2003). Coping strategies as predictors and mediators of disability- related variables and psychosocial adaptation: an exploratory investigation. Rehabil Couns Bull.

[4] Yueh B, Shapiro N, MacLean CH, Shekelle PG (2003). Screening and management of adult hearing loss in primary care: scientific review. J Am Med Assoc.

[5] Gates GA, Mills JH (2005). Presbycusis. Lancet.

[6] Mick P, Kawachi I, Lin FR (2014). The association between hearing loss and social isolation in older adults. Otolaryngol Head Neck Surg.

[7] Cacciatore F, Napoli C, Abete P, Marciano E, Triassi M, Rengo F (1999). Quality of life determinants and hearing function in an elderly population: Osservatorio Geriatrico Campano study group. Gerontology.

[8] Kramer SE, Kapteyn TS, Kuik DJ, Deeg DJH (2002). The association of hearing impairment and chronic diseases with psychosocial health status in older age. J Aging Health.

[9] Gopinath B, Wang JJ, Schneider J, Burlutsky G, Snowdon J, McMahon CM, Leeder SR, Mitchell P (2009). Depressive symptoms in older adults with hearing impairments: the blue mountains study: letters to the editor. J Am Geriatr Soc.

[10] Allen NH, Burns A, Newton V, Hickson F, Ramsden R, Rogers J, Butler S, Thistlewaite G, Morris J (2003). The effects of improving hearing in dementia. Age Ageing.

[11] Livingston G, Sommerlad A, Orgeta V, Costafreda SG, Huntley J, Ames D, Ballard C, Banerjee S, Burns A, Cohen-Mansfield J, Cooper C, Fox N, Gitlin LN, Howard R, Kales HC, Larson EB, Ritchie K, Rockwood K, Sampson EL, Samus Q, Schneider LS, Selbæk G, Teri L, Mukadam N (2017). Dementia prevention, intervention, and care. Lancet.

[12] Fulton SE, Lister JJ, Bush AL, Edwards JD, Andel R (2015). Mechanisms of the hearing-cognition relationship. Semin Hear.

[13] Tilvis RS (2004). Predictors of cognitive decline and mortality of aged people over a 10- year period. J Gerontol- Series A Biol Sci Med Sci.

[14] Wilson RS, Krueger KR, Arnold SE, Schneider JA, Kelly JF, Barnes LL, Tang Y, Bennett DA (2007). Loneliness and risk of Alzheimer disease. Arch Gen Psychiatry.

[15] Fratiglioni L, Wang HX, Ericsson K, Maytan M, Winblad B (2000). Influence of social network on occurrence of dementia: a community-based longitudinal study. Lancet.

[16] WHO (2001) International Classification of Functioning, Disability and Health (ICF). [cited 2018 26.04.18]; Available from: http://www.who.int/classifications/icf/en/

[17] Ewertsen HW, Birk-Nielsen H (1973). Social hearing handicap index. Social handicap in relation to hearing impairment. Audiology.

[18] Mondelli MF, Souza PJ (2012). Quality of life in elderly adults before and after hearing aid fitting. Braz J Otorhinolaryngol.

[19] Niemensivu R, Manchaiah V, Roine RP, Kentala E, Sintonen H (2015). Health-related quality of life in adults with hearing impairment before and after hearing-aid rehabilitation in Finland. Int J Audiol.

[20] Contrera KJ, Betz J, Li L, Blake CR, Sung YK, Choi JS, Lin FR (2016). Quality of life after intervention with a cochlear implant or hearing aid. Laryngoscope.

[21] Kalluri S, Humes L (2012). Hearing technology and cognition. Am J Audiol (Online).

[22] Amieva H, Ouvrard C, Giulioli C, Meillon C, Rullier L, Dartigues JF (2015). Self-reported hearing loss, hearing aids, and cognitive decline in elderly adults: a 25-year study. J Am Geriatr Soc.

[23] Weinstein BE, Spitzer JB, Ventry IM (1986). Test- retest reliability of the hearing handicap inventory for the elderly. Ear Hear.

[24] Miller GA (1956). The magical number seven plus or minus two: some limits on our capacity for processing information. Psychol Rev.

[25] Schow RL, Nerbonne MA (1982). Communication screening profile: use with elderly clients. Ear Hear.

[26] Guyatt GH, Feeny DH, Patrick DL (1993). Measuring health- related quality of life. Ann Intern Med.

[27] Landy FJ (1986). Stamp collecting versus science. Am Psychol.

[28] Demorest ME, Walden BE (1984). Psychometric principles in the selection, interpretation, and evaluation of communication self-assessment inventories. J Speech Hear Disord.

[29] Nunnally JC (1994) Psychometric theory. 3rd edn. In: Bernstein IH. McGraw-Hill, New York

