# Supplementary material for: Understanding the links between hearing impairment and dementia: development and validation of the Social and Emotional Impact of Hearing Impairment (SEI-HI) questionnaire
Source: Neurol Sci. 2020 Jun 10;41(12):3711–7. doi: 10.1007/s10072-020-04492-5 (PMC7655555; doi:10.1007/s10072-020-04492-5)
Supplement: Supplementary file 1 — (DOCX 23 kb) [file 10072_2020_4492_MOESM1_ESM.docx]

**Social & Emotional Impact of Hearing Impairment (SEI-HI) Questionnaire**

The purpose of this questionnaire is to identify any difficulties you are currently experiencing as a result of hearing loss. **Select a number from 1-5 next which corresponds to each statement.**

| 1. **How often do you have communication difficulties when speaking with one other person?** | 1 | 2 | 3 | 4 | 5 |
| --- | --- | --- | --- | --- | --- |
| 1. **How often do you have communication difficulties when speaking in a small group of people?** | 1 | 2 | 3 | 4 | 5 |
| 1. **How often do you have difficulty hearing or communicating in a noisy environment?** | 1 | 2 | 3 | 4 | 5 |
| 1. **Do you lose track of conversations and become embarrassed by the outcome?** | 1 | 2 | 3 | 4 | 5 |
| 1. **Does your hearing situation cause you to become irritable?** | 1 | 2 | 3 | 4 | 5 |
| 1. **How often does your hearing cause you to become frustrated with members of the family?** | 1 | 2 | 3 | 4 | 5 |
| 1. **How often does your hearing cause you to avoid parties or social events?** | 1 | 2 | 3 | 4 | 5 |
| 1. **Does your hearing cause you to feel stupid or dumb?** | 1 | 2 | 3 | 4 | 5 |
| 1. **How often do you have difficulty hearing when somebody speaks in a whisper?** | 1 | 2 | 3 | 4 | 5 |
| 1. **Do you feel handicapped by a hearing problem?** | 1 | 2 | 3 | 4 | 5 |
| 1. **How often do you have difficulty listening to the TV or radio?** | 1 | 2 | 3 | 4 | 5 |
| 1. **Do you feel that any difficulty with your hearing limits or hampers your personal or social life?** | 1 | 2 | 3 | 4 | 5 |
| 1. **How often does your hearing cause you difficulty in a restaurant with relatives and friends?** | 1 | 2 | 3 | 4 | 5 |
| 1. **How often do communication difficulties cause you feel left out in a group of people?** | 1 | 2 | 3 | 4 | 5 |

Do you wear Hearing Aids? YES / NO

If YES, on average day, how long for? ............... hours

On a scale of 1-10 **(1= unsatisfied, 10= most satisfied)** what is your overall satisfaction with the hearing aids?

**1 2 3 4 5 6 7 8 9 10**
